# The complete chloroplast genome sequence of *Corylopsis sinensis* (Hamamelidaceae)

**DOI:** 10.1080/23802359.2022.2044400

**Published:** 2022-02-24

**Authors:** Haoyu Zhang, Jiahao Gu, Hong Chang

**Affiliations:** Key Laboratory of Bio-Resource and Eco-Environment of Ministry of Education, College of Life Sciences, Sichuan University, Chengdu, Sichuan, People’s Republic of China

**Keywords:** *Corylopsis sinensis*, chloroplast genome, phylogenetic analysis

## Abstract

*Corylopsis sinensis* Hemsl. is a deciduous shrub endemic to China, which is a valuable medicinal and ornamental species. In this study, we report the complete chloroplast genome sequence of *C*. *sinensis*, providing a genomic basis for future research. The chloroplast genome is 159,419 base pairs (bp) in length, with a large single-copy (LSC) region of 88,152 bp, a small single-copy (SSC) region of of 18,701 bp, and two inverted repeat (IR) regions of 26,283 bp. The overall GC content is 38.0% and the chloroplast genome encodes 113 unique genes including 79 protein-coding genes, 30 tRNA genes, and 4 rRNA genes. The phylogenetic results show that *C. sinensis* is sister to *C. spicata*. These results of *C. sinensis* will improve our understanding of the evolution of *Corylopsis* and Hamamelidaceae.

*Corylopsis sinensis* Hemsl. is a deciduous shrub of the family Hamamelidaceae (Li et al. 1999). This species is native to China, and mainly distributed in forests or mountains of southern China (Zhang et al. 2003). *C*. *sinensis* is cultivated as an ornamental in gardens and parks due to its beautiful yellow flowers in early spring (Iwashina et al. 2009; Kim et al. 2016;). In addition, the whole plant of *C*. *sinensis* contains bergenin and it is a traditional Chinese medicine for the treatment of chronic bronchitis (Li et al. 2011). Therefore, *C*. *sinensis* has high ornamental and medicinal value. Furthermore, determining the systematic position of *C*. *sinensis* will provide valuable information for the evolution of *Corylopsis* and Hamamelidaceae. As a semi-autonomous organelle, chloroplast play significant role in photosynthesis and physiology of plant (Chang et al. 2021). However, the chloroplast genome of *C*. *sinensis* remains unpublished. Here, we assembled and characterized the complete chloroplast genome of *C. sinensis* to provide valuable genomic resources for further studies.

Fresh leaves of *C. sinensis* were collected from Chengdu Botanical Garden (104°7′42.54″E, 30°45′54.07″N), Sichuan province, China, and the specimen was deposited in Herbarium of Sichuan University (SZ) (http://mnh.scu.edu.cn/, Hong Chang, changhong1123@foxmail.com) under the voucher number SZ02049686. The total genomic DNA was extracted using a modified CTAB method (Doyle and Doyle 1987) from about 0.2 g of leaves. The library with insert size of 300 bp fragments was prepared and sequenced by Illumina platform. The raw reads were first filtered to remove paired-end reads if either of the reads contained (i) adapter sequences, (ii) more than 10% of N bases, and (iii) more than 50% of bases with a Phred quality score less than five. The filtered reads were then assembled using NOVOPlasty version 2.7.2 (Dierckxsens et al. 2017). The assembled chloroplast genome was annotated using Plann version 1.1 (Huang and Cronk 2015). The positions of exons and introns of annotated genes were manually inspected and adjusted by Geneious version 11.0.3 (Kearse et al. 2012) and Sequin version 15.50. (http://www.ncbi.nlm.nih.gov/Sequin/). The annotated chloroplast genome was submitted to GenBank (accession number: MZ590567). To investigate the phylogenetic location of *C. sinensis*, the complete chloroplast genome sequences of *C. sinensis* and ten reported Hamamelidaceae species and two outgroups (*Daphniphyllum pentandrum* Hayata and *Cercidiphyllum japonicum* Siebold & Zucc.) were aligned using MAFFT version 7 software (Katoh and Standley 2013). IQ-tree software (Nguyen et al. 2015) was used to construct maximum likelihood (ML) tree under standard mode with 1000 bootstrap (Anisimova et al. 2011). The nucleotide substitution model was TVM + F+R2 according to the results of ModleFinder (Kalyaanamoorthy et al. 2017). The phylogenetic tree was visualized using online software ITOL V4 (https://itol.embl.de/; Letunic and Bork, 2019).

The complete chloroplast genome of *C. sinensis* is 159,419 base pairs (bp) in length and the GC content is 38.0%. It contains a large single-copy (LSC) region of 88,152 bp, a small single-copy (SSC) region of 18,701 bp, and two inverted repeat sequence (IR) regions of 26,283 bp. It encodes a total of 113 unique genes, including 79 protein-coding genes, 30 tRNA genes, and 4 rRNA genes. The phylogenetic tree indicates that *C. sinensis* is sister to *C. spicata*, and the clade position of genus *Corylopsis* is the sister of genus *Loropetalum* with both a 100% bootstrap support (Figure 1). The complete chloroplast genome of *C. sinensis* and the phylogenetic relationships will enhance our understanding of the evolution of *Corylopsis* and Hamamelidaceae.

**Figure 1.**
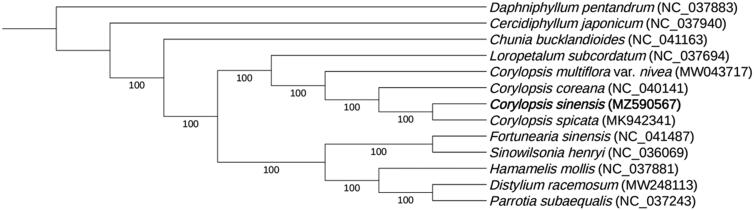
ML phylogenetic tree of *C. sinensis* with 12 previously reported species based on the complete chloroplast genome sequences. Numbers on the nodes are bootstrap values from 1000 replicates.

## Data Availability

The genome sequence data that support the findings of this study are openly available in GenBank of NCBI at (https://www.ncbi.nlm.nih.gov) under the accession no. MZ590567. The associated BioProject, SRA, and Bio-Sample numbers are PRJNA766356, SUB10430244, and SAMN21844338 respectively.
